# Efficacy of 7-Day and 14-Day Triple Therapy Regimens for the Eradication of *Helicobacter pylori*: A Comparative Study in a Cohort of Romanian Patients

**DOI:** 10.1155/2016/5061640

**Published:** 2015-12-27

**Authors:** Stefan Sorin Arama, Catalin Tiliscan, Cristina Negoita, Alexandru Croitoru, Victoria Arama, Carmen Marina Mihai, Florinel Pop, Amit Garg

**Affiliations:** ^1^Pathophysiology and Immunology Department, Dental Medicine Faculty, Carol Davila University of Medicine and Pharmacy, 020022 Bucharest, Romania; ^2^Digestive Endoscopy Department, Dr. Ion Cantacuzino Hospital, 020475 Bucharest, Romania; ^3^Adults III Department, Prof. Dr. Matei Bals National Institute of Infectious Diseases, 021105 Bucharest, Romania; ^4^Personal Genetics Medical Practice, 010987 Bucharest, Romania; ^5^Infectious Diseases I Department, Prof. Dr. Matei Bals National Institute of Infectious Diseases, Carol Davila University of Medicine and Pharmacy, 020022 Bucharest, Romania; ^6^Internal Medicine and Rheumatology Department, Dr. Ion Cantacuzino Hospital, Carol Davila University of Medicine and Pharmacy, 020022 Bucharest, Romania; ^7^Pathology Department, Carol Davila University of Medicine and Pharmacy, 020022 Bucharest, Romania; ^8^Medical Affairs, Dr. Reddy's Laboratories, Hyderabad 500034, India

## Abstract

*Objective*. This study compared the eradication rates of of *Helicobacter pylori* (HP) infection by a 7-day and 14-day anti-HP regimen. *Materials and Methods*. An open, randomized, prospective study was performed to evaluate the response to anti-HP treatment in adult HP-positive patients following a 7-day course (Regimen A) of a proton pump inhibitor in association with clarithromycin and amoxicillin compared to a 14-day course (Regimen B). Gastric biopsies were performed at baseline and two months after anti-HP treatment. *Results*. Seventy-eight patients aged 18–64 years (28 males, 50 females) diagnosed with HP infection were included. Fifty-two (66.7%) patients received Regimen B and 26 (33.3%) Regimen A. The overall eradication rate was 70.5%. Better treatment response (*p* < 0.01) was seen in Regimen B (44/52, 84.2% versus 11/26, 42.3%). Significant improvement in histological features was seen in regimen B. There has been significant overall reduction in endoscopic aspects of gastric and duodenal lesions in both regimens. Younger patients ≤35 years had a better response to Regimen B. Better treatment response was seen in women, urban residents, and those with tertiary level of education in both groups. *Conclusion*. 14-day anti-HP regimen offered a significant better overall eradication of HP in study population.

## 1. Background

Chronic inflammation of gastric mucosa due to* Helicobacter pylori* (HP) infection is associated with the development of dyspeptic symptoms, peptic ulcer disease, and gastric malignancy [1]. Approximately 20% of HP infected people develop gastroduodenal disorders during their lifetime [2]. The prevalence of HP infection is approximately 50% worldwide, depending on geographic area, age, race, and ethnicity, varying between 80% and 90% in developing countries and 35% and 40% in industrialized countries [3].

Symptoms, signs of dyspeptic symptoms, and laboratory tests aid in arriving at a probable diagnosis of HP infection. Though there is no established gold standard for the diagnosis of HP infection, several methods including serology, urea breath test, rapid urease test, faecal antigen test, culture from biopsy, and histological evaluation have good accuracy [4]. Endoscopy helps in assessing the severity of the gastric inflammation caused by HP infection.

Eradication of HP infection substantially reduces the recurrence of associated gastroduodenal diseases. Chronic HP infection can bring in changes in the epithelium of the gastric mucosa resulting in intestinal metaplasia of gastric cells, gastric atrophy, and hypochlorhydria making it more prone to be infected by other organisms [5]. According to European [6] and United States of America guidelines [3], the first-line regimens for treating chronic HP infection in adults consist of a standard triple therapy including a proton pump inhibitor (PPI) with two antibiotics (clarithromycin and amoxicillin or metronidazole) or bismuth-containing quadruple therapy, given for 7–14 days. In geographical areas of high primary resistance to clarithromycin, first-line treatment recommendations include bismuth-containing quadruple therapies, sequential therapy, or nonbismuth-containing quadruple therapy [3, 6]. In clinical practice, seven-day and fourteen-day triple therapy are frequently practiced, each with its own advantages and disadvantages. Varied eradication rates have been observed with seven- versus 14-day regimens [7–9].

The most important causes of treatment failure are patient noncompliance and antimicrobial resistance of the infecting HP strain. Studies suggest that eradication rates achieved by first-line treatment with a PPI, amoxicillin, and clarithromycin have decreased from 94–96% to 70–85%, mostly due to increasing clarithromycin resistance [6].

We conducted this study to compare the efficacy of the triple regimen in the eradication of HP infection in Romanian adults treated for seven and 14 days as there is paucity of data on the treatment outcome. We studied the various factors that may affect the treatment outcome in these patients.

## 2. Objectives

This study was conducted to compare the eradication rates of a seven-day and 14-day anti-HP regimen in a cohort of Caucasian patients. Secondary objectives were to evaluate the overall HP eradication rate and to identify other factors that influenced the treatment success in eradicated patients.

## 3. Materials and Methods

This study was performed in the Medical Department of a Tertiary Care Hospital from Bucharest. Tertiary level or third-level education is defined as the stage of learning that occurs at universities, academies, and institutes of technology. This was an open, randomized, prospective comparative study performed to evaluate the response to anti-HP treatment in HP-positive Caucasian patients.

Patients received either a seven-day treatment regimen (Regimen A) comprising a PPI along with clarithromycin and amoxicillin or a 14-day treatment (Regimen B). Patients of both genders aged 18–64 years with ulcer-like dyspepsia, who met selection criteria and agreed to undergo two endoscopic biopsies, were enrolled after obtaining written informed consent. Alcohol intake of at least one alcoholic beverage/week was noted. HP status was assessed by histology and urease rapid test. Patients with systemic, autoimmune, metabolic, and cardiovascular diseases, severe mental disorders, and cancer (gastric or otherwise), on chronic nonsteroidal anti-inflammatory drugs (NSAIDs), receiving antibiotic treatment in the last 6 months or PPI treatment in the last 2 months, were excluded from the study. The protocol was approved by the local research ethics committee.

HP-positive patients were randomized to receive lansoprazole (Lanzap, Dr. Reddy's Laboratories Ltd.) 30 mg bid, amoxicillin (Ospamox, Novartis Pharma GMBH) 1 g bid, and clarithromycin (Klacid, Abbott) 2 × 250 mg bid (LAC) regimen for either seven or 14 days. Gastric biopsies of antrum and body were performed at baseline and two months after completing the HP eradication treatment. The severity of gastric inflammatory lesions was measured by grading, which represents the semiquantitative assessment of mononuclear and granulocytic infiltrate in antral and corpus biopsy samples, according to the Updated Sydney System [10]. Presence of HP, the degree of chronic inflammation, acute inflammatory activity, and atrophy were analysed.

The gastric biopsies were fixed in buffered 8% formalin solution for 24 hours and then processed by standard histopathological techniques (embedding in paraffin wax). Three micrometer sections were obtained and were stained by hematoxylin and eosin (H&E), Giemsa, Alcian yellow, and immunohistochemistry (IHC). The IHC technique used a mouse IgG1 monoclonal antibody, clone BC7 Biocare (USA), having the IVD/FFPE standard [11].

A negative rapid urease test and absence of the bacteria from gastric biopsies were the criteria to prove HP eradication.

All statistical calculations were performed by using the statistical package SPSS.15 (SPSS Inc., Chicago, IL). Numeric variables were analyzed with Mann-Whitney test. Correlations between response to treatment data and variables that could influence it were analyzed with Chi-square test. The level of significance was set at *p* < 0.05.

## 4. Results

Seventy-eight patients (age: 18–64, median: 40 years) with symptoms of ulcer-like dyspepsia, including 28 men (35.9%, age range: 22–64 years, median: 41.5 years) and 50 women (64.1%, age range: 18–63 years, median: 37.5 years), diagnosed with HP infection by rapid urease test and histology, were included in the study.  Table 1 illustrates demographic, clinical, and histological data of study patients. All patients had dyspeptic symptoms and their endoscopy revealed various grades of oesophagitis, gastritis, and duodenitis (Table 2).

Fifty-two (66.7%) patients received 14-day and 26 (33.3%) received 7-day triple therapy. There were nine men and 17 women in 7-day treatment. In the 14-day treatment group, there were 19 men and 33 women and there was no statistical significance of gender variation between groups. Statistically significant differences between two groups were noted in median age (*p* < 0.05) and the level of education (*p* = 0.01) with 26 having their tertiary level of education in 14-day treatment. Alcohol intake was more in 14-day treatment group (27/35), statistically significant (*p* = 0.04). Of 32 smokers, 22 were in 14-day treatment which was of no statistical significance.

Overall eradication rate observed was 70.5% (55/78). The number of patients who responded to treatment was significantly greater in the 14-day treatment (84.6%, 44/52) group compared to patients who received the 7-day treatment group (42.3%, 11/26) (*p* < 0.001). Eradication of* H. pylori* was seen in 55 patients of whom 44 were in 14-day treatment group.

Eradication was seen in 22 men (five in duration of 7 days) and in 33 females (six in 7-day treatment), but there is no statistically significant difference based on gender. Eradication rates were better in nonalcoholics, nonsmokers, women, urban residents, those with tertiary level of education.

After treatment, no statistically significant changes were seen in oesophagitis between the two treatment groups (7.7%). After treatment, statistically significant difference (*p* < 0.05) was seen in gastritis within treatment groups; however, there was no significant change between the groups after treatment. There was no statistically significant change in duodenitis between both treatment groups; however, statistically significant (*p* < 0.05) changes were seen in 14-day treatment compared to 7-day treatment. After treatment, there was no statistically significant change in gastric and duodenal ulcer between both treatment groups.

**Figure 1:**
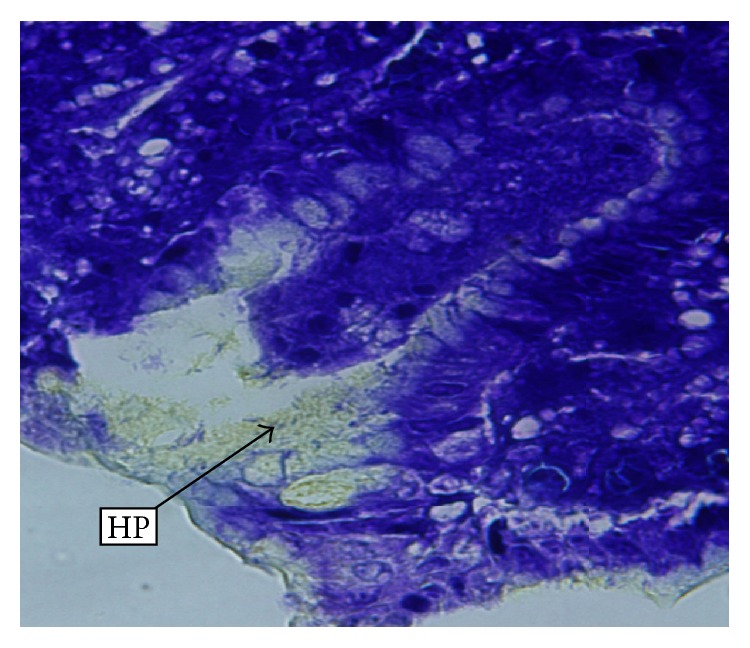
Vacuolising lesions of the foveolar epithelium. Numerous HP (*H. pylori*) colonies are visible in the foveolar mucus. (Alcian yellow stain; 40x magnification.)

**Figure 2:**
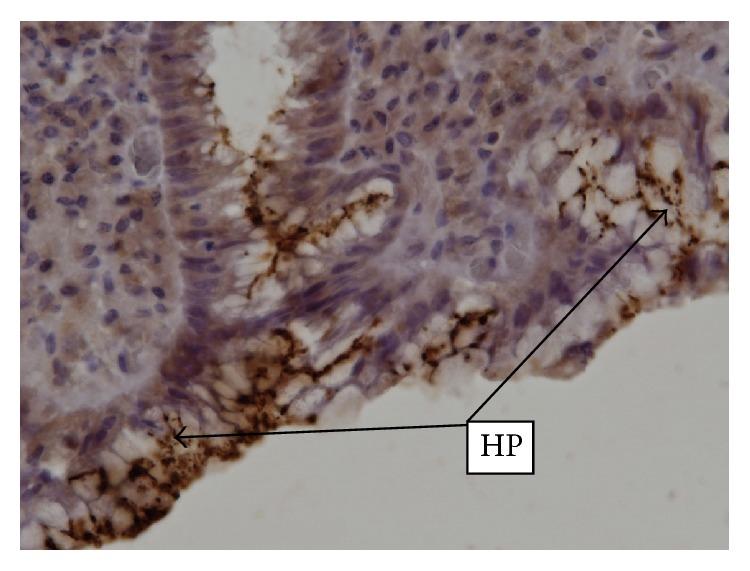
Numerous HP colonies on the surface of the foveolar epithelium. Epithelial cells present marked vacuolising lesions and destruction of the apical pole, giving the impression of intracellular HP invasion. (IHC, ACMo Anti-HP Biocare, clone BC 7; 40x magnification.)

**Figure 3:**
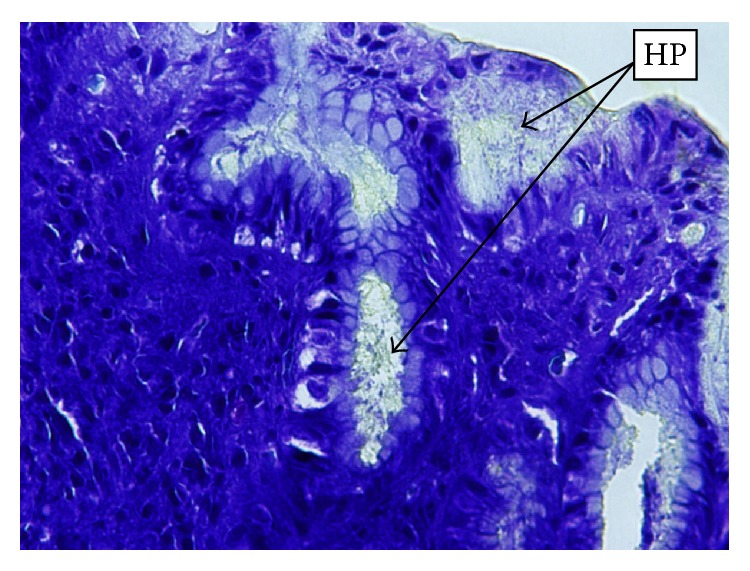
Healing processes after 7-day therapy: marked reduction of vacuolising lesions, restoring of foveolar architecture, reduction of inflammatory reaction, and persistence of HP colonies. (Alcian yellow; 20x magnification.)

Significant improvement in histological features was seen in patients who received 14-day treatment and none of these patients maintained a high histologic activity index. Normal endoscopic features were seen in 13 patients, of whom five were in 7-day and eight in 14-day treatment.  Figure 1 shows vacuolising lesions and lysis of the foveolar cells, induced by CagA positive HP strains. HP colonies are visible in the foveolar mucus. In patients with important vacuolising lesions and high HP density, typical aspects of pseudocolonisation of intracellular mucus from the apical region of foveolar cells may be observed (Figure 2). In Figure 3, we present a biopsy from a patient who received the 7-day regimen. The acute inflammatory response almost disappeared, and the chronic inflammation was markedly reduced, but HP colonies may still be observed.

## 5. Discussion and Conclusion

Evidences indicate a strong association between HP infection and dyspeptic symptoms, gastric ulcer, duodenal ulcer, and development of gastric cancer. Hence, treating HP infection is essential to reduce the risk of development of these complications including gastric cancer.

According to current guidelines, triple combination therapy is considered as a standard regimen for the first-line anti-HP treatment. There are still debates on the ideal duration of treatment. Available data suggest that the 7-day triple therapy is not inferior to the 14-day therapy and that extending the duration of PPI-clarithromycin containing triple therapy from 7 to 14 days improves HP eradication by only 5% [12]. However, in our study, the better treatment response was significant in the 14-day regimen group (84.6% versus 42.3%).

Studies suggest that eradication rates achieved by first-line treatment with a PPI, amoxicillin, and clarithromycin have decreased to 70–85%, due to increasing clarithromycin resistance. In our study, the overall eradication rate of 70.5% was lower than expected and was definitely influenced by the low success rate of the 7-day regimen. The 14-day regimen provided an acceptable eradication rate (84.6%); however, we must regard these results with caution, due to the relatively small size of the study population. Prospective surveillance studies of HP antibiotic resistance in Romania are required to guide the choice of first-line eradication regimens.

Various studies have noted that HP eradication was significantly associated with age. Mamori et al. [13] reported in 2010 that, of all general factors examined in a cohort of 253 Japanese patients, only age < 50 years was significantly associated with a poor response in HP eradication. Treatment side effects were more common in younger patients, which might explain the lower rate of eradication. Interestingly, we observed HP eradication in 24/25 patients aged ≤ 35 years and in 20/27 patients aged ≥ 35 years in those who received 14-day treatment. No significant age dependent therapeutic response was in patients who received seven-day treatment. These observations indicate that younger patients had a significantly better response with 14-day treatment (24 versus 3), a finding that does not concur with results of study by Mamori et al. Study by Moayyedi et al. [14] has shown that treatment response was better in men. But we have observed better treatment response in females (51.9%) who received 14-day treatment whereas there was no statistically significant gender difference observed in seven-day treatment. We also observed that urban residents responded to the treatment better than those from rural areas. We speculate that variables associated with HP eradication may differ according to race and ethnicity which should be explored and confirmed by further studies.

Data is not available to support the influence of education in HP eradication. We observed that patients with higher education responded significantly better to anti-HP treatment, which may be attributed to better treatment compliance.

Endoscopical findings too indicated better treatment response with 14-day treatment in our study. Both of the two patients who had stage B oesophagitis in 7-day treatment moved to stage A post treatment indicating improvement. In 14-day treatment, of two patients in stage A prior treatment, one was cured and one remained in stage A after treatment; of four patients in stage B prior treatment, three moved to stage A and one was cured after treatment.

There has been significant overall reduction in gastritis and duodenitis in both treatment regimens; however, erythematous gastritis and duodenitis were seen more with seven-day therapy, after treatment. This could be attributed to the reduction in more severe forms of gastritis (erosive and exudative forms) to milder erythematous form.

Among those who received 7-day treatment, two patients, who had erosive gastritis initially, moved to the stage of erythematous gastritis indicating reduction in severity. Of 10 patients with exudative gastritis initially, five were found to have no gastritis after treatment, four patients moved to erythematous gastritis, and status in one patient did not change. In 14-day treatment, of the four patients who had erosive gastritis, one patient was cured completely, two moved to erythematous gastritis, and one moved to exudative gastritis stage. Of the eight patients in exudative gastritis, one was cured completely, six moved to erythematous gastritis, and no change was seen in one patient. Of 39 patients, who had erythematous gastritis, 20 were cured and 19 remained the same.

Lesions in all regions responded well to the treatment but, at the end of therapy, lesions still persisted in the antral region, which could be due to the large number of lesions in the antral region prior treatment. Among 7-day treatment group, of eight pangastric cases, one remained the same, three had antral gastritis, and four were cured. The patient who had corporal gastritis developed antral gastritis. Of 13 antral cases, nine remained the same and four were completely cured.

Among 14-day treatment, of five patients who had pangastric gastritis, two were completely cured and three had antral lesions. Of the two patients with corporal gastritis, both presented antral gastritis post treatment. Of 44 patients who initially had antral gastritis, no change was seen in 23 patients, 20 were cured, and one developed pangastric gastritis.

In 7-day treatment, of the four erosive duodenitis patients, two were cured completely, one moved to erythematous Gastritis/duodenitis, and one moved to exudative duodenitis. Of the five patients in/with exudative duodenitis, one remained the same, one moved to erythematous gastritis/duodenitis, and three were cured. In 14-day treatment, of nine patients with erosive duodenitis, one remained the same, one moved to exudative duodenitis, and seven were cured. Of five exudative duodenitis, four were cured and one remained the same.

Major concern in the treatment of* H. pylori* is increase in the emergence of primary bacterial resistance throughout the globe to the antibiotics used in the triple therapy causing a major hindrance in the treatment [15, 16]. Bacterial resistance especially to clarithromycin which is one of the components of triple regimen is the challenge faced by the physicians as dose increase does not overcome the resistance [17]. In 1998, Glupczynski et al. have noted the global resistance to clarithromycin as 9.9% [18]. In 2010, de Francesco et al. have observed in their study it as 17.2%, with highest rate being reported from North America (29.3%). This study also highlights the problem of increase in the rate of the resistance towards clarithromycin [19]. Camargo et al. have observed the resistance to clarithromycin to be 12% in Latin America [20].

It is difficult to correlate our findings with HP resistance to antibiotics in Romania, because studies on this topic are very few. The results of a research grant conducted in the western region of Romania 2006–2008 showed 100% sensibility to amoxicillin and erythromycin, 33% resistance to clarithromycin, and 40% resistance to metronidazole, but only 10 strains were tested [21]. Ilie et al. have noted the resistance to various antibiotics used in* H. pylori* induced dyspepsia from 100 Romanian patients. They observed that bacterial resistance was high and it was 32% for clarithromycin, 92.8% for metronidazole, and 50% for amoxicillin [22].

The major limitation of this study was that patients undergoing endoscopy for HP-related symptoms constituted a minority of infected population. HP-resistance status in this population may not be representative of the total population with underlying HP infection. Another limitation of our study was the small number of participants but still is representative of the population.

The correlation between histopathological aspects and HP-specific IHC tests represents in our opinion a good option for assessment of the changes induced by HP infection in the gastric mucosa (repair type, atrophy grade, intensity of the inflammatory, and immune response) and for quantification of the therapeutic response.

In conclusion, the 14-day anti-HP regimen offered better HP eradication in our study group, compared to a similar, 7-day regimen. As eradication of symptomatic HP infection substantially reduces the recurrence of associated gastroduodenal diseases and development of gastric cancer, future studies are needed to evaluate these factors influencing treatment success and HP resistance in larger group of Romanian patients and to guide the choice of therapy. In addition, physician should emphasize the influence of alcohol intake and smoking and importance of reducing stress levels in these patients; patient education on the preventive measures and treatment compliance would result in better treatment outcome. However, we recommend further studies to support our findings in a larger population.

## Figures and Tables

**Table 1:** Comparison of demography, disease characteristics, and outcome among Regimen A and Regimen B.

Characteristics	Seven-day treatment (Regimen A) *n* (%)	14-day treatment (Regimen B) *n* (%)	Total *n* (%)	95% confidence interval
Demography				
Number of patients	26 (33.3%)	52 (66.7%)	78	NA
Male : female	9 : 17	19 : 33	28 : 50	NA
Mean age	45	38	40	18–64
Tertiary level of education	6 (23.1%)	26 (50.0%)	32 (42.7%)	NA
Alcohol intake	8 (30.8%)	27 (51.9%)	35 (46.7%)	NA
Smoking	10 (38.5%)	22 (42.3%)	32 (42.7%)	NA
Urban residency	21 (80.8%)	44 (84.6%)	65 (85.9%)	NA
Rural residency	5 (19.2%)	6 (11.5%)	11 (14.1%)	NA
Disease characteristics				
High histologic activity			18 (23.7%)	NA
After treatment				
Eradication of *H. Pylori* ^*∗*^	11 (42.3%)	44 (84.6%)	55 (70.5%)	NA
Treatment response in men	05 (19.2%)	17 (32.7%)	22	NA
Treatment response in women	06 (23.1%)	27 (51.9%)	33	NA
Treatment response in tertiary level education	04 (15.4%)	23 (44.2%)	27	NA
Treatment response in those with lower level education	7 (26.9%)	19 (36.5%)	26	NA
Treatment response in alcohol consumers	03 (11.5%)	23 (44.2%)	26	NA
Treatment response in smokers	03 (11.5%)	21 (40.4%)	24	NA
Treatment response in rural residents	01 (3.8%)	05 (9.6%)	06	NA
Treatment response in urban residents	10 (38.5%)	38 (73.1%)	48	NA
Normal endoscopy	05 (19.2%)	08 (15.4%)	13	NA

^*∗*^ *P* < 0.05.

**Table 2:** Endoscopic features before and after treatment in both groups.

	Seven-day regimen		14-day regimen	
	Pretreatment number (%)	Posttreatment number (%)	Pretreatment number (%)	Posttreatment number (%)
Number of patients	26 (33.3%)	26 (33.3%)	52 (66.7%)	52 (66.7%)
Oesophagitis	2 (7.7%)	2 (7.7%)	6 (11.5%)	4 (7.7%)
Stage A	0 (0%)	02 (7.7%)	2 (3.8%)	04 (7.7%)
Stage B	02 (7.7%)	0 (0%)	4 (7.7%)	0 (0%)
Gastritis	22 (84.6%)	15 (57.7%)	51 (98.1%)	29 (55.8%)
Erythematous gastritis	10 (38.5%)	14 (53.8%)	39 (75.0%)	28 (53.8%)
Exudative gastritis	10 (38.5%)	01 (3.8%)	08 (15.4%)	01 (1.9%)
Erosive gastritis	02 (7.7%)	0 (0%)	04 (7.7%)	0 (0%)
Site of gastritis				
Antral	13 (50.0%)	14 (53.8%)	44 (84.0%)	28 (53.8%)
Corporal	01 (3.8%)	0 (0%)	02 (3.8%)	0 (0%)
Pangastric	08 (30.8%)	01 (3.8%)	05 (9.6%)	01 (1.9%)
Duodenitis	9 (34.6%)	5 (19.2%)	14 (26.9%)	3 (5.8%)
Erythematous duodenitis	0 (0%)	02 (7.7%)	0 (0%)	0 (0%)
Exudative duodenitis	05 (19.2%)	03 (11.5%)	05 (9.6%)	02 (3.8%)
Erosive duodenitis	04 (15.4%)	0 (0%)	09 (17.3%)	01 (1.9%)
Gastric ulcer	3 (11.5%)	1 (3.8%)	1 (1.9%)	0 (0%)
Duodenal ulcer	4 (15.4%)	1 (3.8%)	2 (3.8%)	1 (1.9%)
